# The Ca^2+ ^activated SK3 channel is expressed in microglia in the rat striatum and contributes to microglia-mediated neurotoxicity *in vitro*

**DOI:** 10.1186/1742-2094-7-4

**Published:** 2010-01-14

**Authors:** Lyanne C Schlichter, Vikas Kaushal, Iska Moxon-Emre, Vishanthan Sivagnanam, Catherine Vincent

**Affiliations:** 1 Genes and Development Division, Toronto Western Research Institute, University Health Network, Toronto, Ontario, M5T 2S8, Canada; 2 Department of Physiology, University of Toronto, Toronto, Ontario, M5S 1A8, Canada; 3 Current address: 735 1re (Premiere) Avenue, Unit 205, Lachine, Quebec, H8S 2S6, Canada; 4 Current address: Technology Development and Commercialization, University Health Network, 101 College Street, Suite 150, Toronto, Ontario, M5G 1L7, Canada; 5 Corresponding address: MC9-417; Toronto Western Hospital, 399 Bathurst Street, Toronto, Ontario, M5T 2S8, Canada

## Abstract

**Background:**

Small-conductance Ca^2+ ^activated K^+ ^channels are expressed in the CNS, where *KCNN2*/SK2/KCa2.2 and *KCNN3*/SK3/KCa2.3 help shape the electrical activity of some neurons. The SK3 channel is considered a potential therapeutic target for diseases and disorders involving neuron hyper-excitability but little is known about its expression and roles in non-neuronal cells in either the healthy or damaged CNS. The purpose of this study was to examine expression of *KCNN3*/SK3 in CNS microglia *in vivo *and *in vitro*, and to use an established *in vitro *model to determine if this channel contributes to the neurotoxic capacity of activated microglia.

**Methods:**

*KCNN3 *mRNA (real-time RT-PCR) and SK3 immunoreactivity were examined in rat microglia. Lipopolysaccharide was then used to activate microglia (monitored by iNOS, nitric oxide, activation of NF-κB and p38 MAPK) and transform them to a neurotoxic state. Microglia-mediated neuron damage (TUNEL, activated caspase 3) and nitrotyrosine levels were quantified using a two-chamber system that allowed microglia to be treated with channel blockers, washed and then added to neuron/astrocyte cultures. Contributions of SK3 to these processes were discriminated using a subtractive pharmacological approach with apamin and tamapin. ANOVA and post-hoc tests were used to assess the statistical significance of differences between treatment groups. SK3 immunoreactivity was then compared in the normal and damaged adult rat striatum, by injecting collagenase (a hemorrhagic stroke) or endothelin-1 (a transient ischemic stroke).

**Results:**

*KCNN3 *mRNA was prevalent in cultured microglia and increased after lipopolysaccharide-induced activation; SK3 channel blockade inhibited microglial activation and reduced their ability to kill neurons. SK3 immunoreactivity was prevalent in cultured microglia and throughout the adult rat striatum (except white matter tracts). After strokes, SK3 was highly expressed in activated microglia/macrophages within the lesions, but reduced in other cells.

**Conclusions:**

SK3 is expressed in microglia in both the healthy and damaged adult striatum, and mechanistic *in vitro *studies show it contributes to transformation of microglia to an activated neurotoxic phenotype. Thus, SK3 might be a therapeutic target for reducing inflammation-mediated acute CNS damage. Moreover, its roles in microglia must be considered when targeting this channel for CNS diseases, disorders and reducing neuron hyper-excitability.

## Background

After acute CNS injuries such as stroke or trauma, there is a prolonged inflammatory response involving microglial activation and infiltration of macrophages and neutrophils, which has the potential to cause secondary injury [1-4]. Contributions of activated microglia are complex because they can produce cytotoxic molecules, as well as growth and repair factors [5-9]. There is a need to identify drug targets for reducing detrimental outcomes of inflammation without interfering with beneficial microglial functions, such as phagocytosis, which removes cellular debris, aids in repair and facilitates reorganization of neuronal circuits [10,11]. Microglia respond to CNS damage by up-regulating functions that involve Ca^2+ ^signaling; e.g., proliferation, migration, phagocytosis, and production of nitric oxide, interleukins, cytokines and chemokines [12-17]. An anticipated immediate response to a rise in intracellular Ca^2+ ^is activation of small- and intermediate-conductance Ca^2+^-activated K^+ ^channels, which are very sensitive to Ca^2+ ^increases and do not require changes in membrane potential to activate [18-20]. Intermediate-conductance Ca^2+^-activated K^+ ^channels (variously called *KCNN4*/KCa3.1/SK4/IK) have been described in microglia [21-23], but very little is known about other SK channels or their roles in these cells.

There are three mammalian SK channels (*KCNN1*/SK1, *KCNN2*/SK2, *KCNN3*/SK3), which are predominantly expressed in the nervous system. By contributing to the medium duration after-hyperpolarization, SK channels regulate neuronal excitability, phasic firing patterns and action potential propagation [24-26]. The SK3 channel is under increasing scrutiny because of its expression patterns in neurons in the caudate putamen, hippocampus and dorsal motor nucleus, its role in action potential firing in dopaminergic neurons, and the possible links between several CNS disorders and SK3 mutations or changes in expression (see Discussion). The resulting impetus to develop SK3 inhibitors, activators and modulators as therapeutic tools makes it essential to understand the roles of this channel in other cells, both within the CNS and in peripheral tissues. Very little is known about the expression, and particularly, the roles of SK channels in non-neuronal CNS cells; this study addresses the contribution of these channels to activation and potentially cytotoxic functions of microglia.

First, we compared transcript expression of *KCNN1*, *KCNN2 *and *KCNN3 *in microglia isolated from rat brain. *KCNN3 *predominated in unstimulated microglia, and they had substantial SK3 immunoreactivity. *KCNN3 *was selectively increased in microglia that were activated by lipopolysaccharide, which increases intracellular Ca^2+ ^[27,28] and up-regulates pro-inflammatory molecules. Activated microglia killed neurons *in vitro*; and the use of potent peptide channel blockers demonstrated a specific role for SK3 in microglial activation through p38 MAP kinase, and in nitric oxide production and neurotoxicity. Importantly, NFκB activation and phagocytosis were not inhibited. These results provide the first evidence that blocking SK3 channels in microglia can reduce their cytotoxicity, but does not globally inhibit their functions. To demonstrate that these findings are relevant to the adult CNS, we showed SK3 immunoreactivity in microglia in the healthy rat striatum, with substantial expression in activated microglia and macrophages after both ischemic and hemorrhagic strokes.

## Methods

All procedures on animals were approved by the University Health Network animal care committee, in accordance with guidelines established by the Canadian Council on Animal Care. All rats were obtained from Charles River, St.-Constant, PQ. Prior to inducing a stroke, adult male Sprague-Dawley rats (250-280 g; 3-4 months old) were housed in pairs, maintained under a 12 h light/dark cycle, and given food and water *ad libitum*. For preparing microglia cultures, 1-2 day-old rat pups were killed by cervical dislocation.

### Cell cultures

Microglia (≥99% pure) cultures were prepared using our standard protocols [22,29,30]. After removing the meninges from 1-2 day old rats, the brain was dissected, minced in cold Minimal Essential Medium (MEM; Invitrogen, Carlsbad CA), centrifuged (300 g, 10 min) and re-suspended in MEM supplemented with 5% horse serum and 5% fetal bovine serum (Wisent, St-Bruno, PQ), and 0.05 mg/ml gentamycin (Invitrogen). Two days later, cellular debris, non-adherent cells and supernatant were removed, fresh medium was added to the flask and the mixed cultures were allowed to grow for another 8-10 days. Microglia suspensions were harvested by shaking the flasks on an orbital shaker (65 rpm, 4-6 h, 37°C), and then seeded in MEM with 2% fetal bovine serum. Neuron cultures were prepared from E18 rat embryos, as before [22,29]. After removing the meninges and brain stem, the tissue was isolated, incubated in 2 mg/ml papain (Worthington Biochemical, NJ) for 30 min at 37°C, and triturated in Neurobasal A/B27 medium. The neuron layer was re-suspended in antioxidant-free Neurobasal A medium with 2% B27 supplement, 0.05 mg/ml gentamycin and 0.5 mM L-glutamine (all from Invitrogen). Neurons were seeded on poly-L-ornithine-treated German coverslips (Bellco Glass Inc., Vineland, NJ) at 3 × 10^4 ^cells/well and grown for 7-10 days to increase the proportion of mature neurons [22]. Every 4 days, 50% of the medium was replaced with fresh antioxidant-free Neurobasal A/B27. For studies of microglia-mediated neurotoxicity, microglia were seeded in the same medium used for neurons. When used to validate the SK3 immunostaining, non-transfected and stably transfected (with rSK3) CHO cells were grown in Iscove's Modified Dulbecco's Medium with 10% fetal bovine serum, HT supplement, antibiotic-antimycotic, and 0.4 μg/ml geneticin (G418) (all from Gibco).

### Induction of a hemorrhagic or ischemic stroke

An intracerebral hemorrhage (ICH) was induced in the striatum of adult male Sprague-Dawley rats (300-350 g), as before [31-35]. In brief, rats were anesthetised with isofluorane (3% induction, 1.5% maintenance) and placed in a stereotaxic frame. A 1 mm diameter burr hole was drilled in the skull (0.2 mm anterior and 3 mm lateral to bregma), through which a 30-gauge needle was lowered into the right caudate putamen. A micropump (Micro4, World Precision Instruments, Sarasota, FL) delivered 0.125 U of bacterial type IV collagenase (Sigma-Aldrich, Oakville, ON) in 0.5 μl saline, at 250 nl/min. This resulted in a hemorrhagic lesion that was limited to the striatum. To induce an ischemic stroke in the striatum, the same surgical procedures were used, but the vasoconstrictor peptide, endothelin-1 (Calbiochem, EMD Biosciences; San Diego, CA) was injected (400 pmol in 1.0 μl of saline). This widely used model produces a transient focal ischemic stroke [36], which we found was of reproducible size and restricted to the striatum [37]. After each injection, the needle was left in place for 5 min to prevent solution reflux. The animal's core body temperature was maintained at 37.0 ± 0.5°C using an electric heating pad throughout surgery and recovery, and rats regained consciousness within 10 min.

### Immunohistochemical analysis

All antibodies were diluted in 2.5% donkey serum and centrifuged before use at 9400 g for 10 min to remove any aggregated antibody. The concentrations used for primary and secondary antibodies are indicated in the figure legends. Cultured microglia were plated on glass coverslips and grown for 3 days in MEM with 2% fetal bovine serum and 0.05 mg/ml gentamycin. After washing with PBS (3×, 5 min each), they were fixed for 30 min in 4% paraformaldehyde, washed (3×, 5 min each), permeabilized for 5 min with 0.2% Triton X-100, and washed again (3×, 5 min each). SK3 immunoreactivity was detected with an anti-SK3 antibody (rabbit polyclonal, Alomone Labs, Jerusalem, Israel) that we previously validated by Western blots on microglia and rSK3-transfected CHO cells [21]. Non-specific antigens were blocked with 4% donkey serum for 1.5 h and microglia were incubated with the anti-SK3 antibody overnight at 4°C. After washing, cells were incubated with a donkey anti-rabbit secondary antibody (Jackson ImmunoResearch Laboratories) and microglia were co-labeled (50 min, room temperature) with tomato lectin (FITC-conjugated TL, Sigma-Aldrich), which binds to microglial cell surface N-acetyl-glucosamine residues and to N-acetyl-lactosamine on lysosomes of activated cells. After further washing (PBS, 3×, 10 min each), cell nuclei were labeled for 5 min with DAPI (4'-6-diamidino-2-phenylindole) (1:3000; Sigma-Aldrich). The coverslips were washed (PBS, 3×, 5 min each) and mounted on glass slides with VectaShield mounting medium (Vector Labs, Burlingame, CA). Microglia and CHO cells were imaged with an Axioplan 2 deconvolution microscope, 60× quartz objective, Axiocam HRm digital camera, and analyzed with Axiovision 4.6 software (all from Zeiss).

After a stroke or ICH, rats were killed by an overdose of isofluorane, and then perfused through the heart with 100 ml phosphate buffered saline (PBS), followed by 60 ml of fixative (4% paraformaldehyde, 2% sucrose in PBS; pH 7.5). Dissected brains were stored in the same fixative at 4°C overnight, followed by 10% sucrose for 24 h and 30% sucrose for 48 h. Fixed brains were cut coronally through the needle entry site, and at 4 mm anterior and 4 mm posterior to that plane. Frozen brain sections (16 μm thick) were made using a Model CM350S cryostat (Leica, Richmond Hill, ON) and stored at -40°C until used. Several antibodies were used to illustrate the salient features of the striatum, ischemic and hemorrhagic lesions, microglia and macrophages, as we recently used [32-35]. White matter tracts were labeled with an antibody against myelin basic protein (MBP) (mouse monoclonal, Sigma-Aldrich), one of the most abundant proteins in the myelin sheath. Three different labels were used for microglia and macrophages: an antibody against rat CD11b/c, which is also called complement receptor 3 (OX-42; mouse monoclonal, AbD Serotec, Oxford, UK), an antibody against 'ionized calcium-binding adapter-1' (Iba-1; rabbit polyclonal, Wako, Osaka, Japan), or tomato lectin (as above). The choice of label was based on staining clarity, which depended on the tissue and cell activation state, and on compatibility; i.e., polyclonal rabbit Iba-1 and SK3 antibodies could not be combined. The cryosections were incubated (24 h, 4°C) with a primary antibody in PBS containing 3% donkey serum and 0.3% Triton X-100, and then washed in PBS (3×, 5 min each). Fluorophore-conjugated secondary antibodies were donkey anti-rabbit or anti-mouse, as appropriate (see figure legends), applied for 2 h at room temperature in PBS containing 3% donkey serum and 0.3% Triton X-100. After the sections were washed (PBS, 2×, 10 min each), they were cover-slipped using an aqueous mounting medium (1:1 glycerol:PBS). Negative controls were treated in the same manner, without the primary antibody. Sections were examined using an LSM 510 META confocal microscope (Zeiss, Oberkochen, Germany) and the final images were prepared using ImageJ software (version 1.33k, NIH).

### Quantitative real-time reverse transcriptase polymerase chain reaction (qRT-PCR)

qRT-PCR [38] was used to monitor transcript expression of SK channels in cultured microglia, as we have previously done for other ion channels [22,30]. Gene-specific primers were designed with the 'Primer3Output' program http://frodo.wi.mit.edu/primer3/input.htm, as follows. ***KCNN1 *(SK1) **(GenBank #AF000973): forward 5'-TGG ACA CAC AGC TCA CCA A-3'; reverse 5'-ATG GAT GGC CTG AAG GAA C-3'. ***KCNN2 *(SK2) **(GenBank #NM_019314): forward 5'-ACT TCC TTG GAG CAA TGT GG-3'; reverse 5'-TGC ACA TGC TTT TCT GCT TT-3'. ***KCNN3 *(SK3) **(GenBank #U69884) forward 5'-CCA ACC CCT CCA GCT CTT-3'; reverse 5'-GTT GGC TTT GGG GAA GGT-3'. **TBP **(TATA box binding protein) (GenBank #XM_217785): forward 5'-GGA CCA GAA CAA CAG CCT TC-3'; reverse 5'-CCG TAA GGC ATC ATT GGA CT-3'. RNeasy mini kits (Qiagen, Mississauga, ON) were used to isolate RNA after degrading any contaminating DNA with DNaseI (0.1 U/ml, 15 min, 37°C; Amersham Biosciences, Baie d'Urfe, PQ). A two-step reaction was performed according to the manufacturer's instructions (Invitrogen); i.e., total RNA (2 μg) was reverse transcribed in 20 μl volume using 200 U of SuperScriptII RNase H-reverse transcriptase, with 0.5 mM dNTPs (Invitrogen) and 0.5 μM oligo dT (Sigma-Aldrich). Amplification was performed on an ABI PRISM 7900 Sequence Detection System (PE Biosystems, Foster City, CA) at 95°C for 10 min, followed by 40 cycles at 95°C for 15 s, 55°C for 15 s and 72°C for 30 s. 'No-template' and 'no-amplification' controls were included for each gene, and melt curves showed a single peak, confirming specific amplification. Relative input RNA amounts were determined from a relative standard curve for each gene of interest and the housekeeping gene, TBP.

### Microglia stimulation, with and without SK blockers

Lipopolysaccharide (LPS) is a cell wall component of gram-negative bacteria that binds to Toll-like receptor 4 and is commonly used to activate microglia *in vitro *[39,40]. We compared control microglia and those treated with LPS from the commonly used *E. coli *strain, 0055:B5 (Sigma-Aldrich), with or without a channel blocker. (Figure legends indicate concentrations of LPS and blockers for each experiment.) A pharmacological approach was used to investigate the roles of SK channels because microglia, like other innate immune cells (macrophages, neutrophils), are extremely resistant to transfection, infection or siRNA-mediated knockdown. Because no specific SK3 inhibitors are available, we designed a subtractive protocol to unequivocally discriminate roles of SK3 channels, based on affinities of apamin and tamapin for cloned SK channels [26]. IC_50_'s of apamin are 27-140 pM for SK2, 0.6-4.0 nM for SK3, and 0.7-12 nM for recombinant human SK1; thus, we first tested a relatively high concentration of apamin. If 100 nM apamin (Alomone) blocked a microglial function, then we compared a high and low concentration of the scorpion toxin, tamapin. If apamin affected the function of interest, then 5 nM tamapin (Alomone) was used, which fully blocks SK2 (K_d _= 24 pM) and significantly blocks SK3 (K_d _= 1.7 nM) [41]. Finally, if 5 nM tamapin was effective, then 250 pM tamapin was used to preferentially block SK2 channels, if present. Thus, a role for SK3 channels was deduced if a cellular function was inhibited by apamin and 5 nM tamapin, but not by 250 pM tamapin.

### Microglia-mediated neuron damage

To assess whether SK3 channels contribute to microglia-mediated damage to neurons, we used a Transwell™ system (BD Biosciences, Franklin Lakes, NJ), as before [22]. Microglia in the porous upper inserts (10^6 ^cells/insert) were incubated overnight (95% O_2_, 5% CO_2_, 37°C), and then activated with LPS for 24 h, with or without apamin or tamapin. Neuron/astrocyte cultures (~70:30%) were grown on a coverslip in the bottom well of the chamber. The microglia-bearing inserts were then washed thoroughly with Neurobasal A medium (Invitrogen), so that the target neurons were never exposed to drugs, and then placed above healthy neuron/astrocyte cultures and incubated for 24 or 48 h, depending on the assay. The 3-μm diameter pores in the upper insert allow the microglial cells to chemically communicate, without cell-cell contact.

For analysis, the neuron-bearing coverslips were removed from the chamber, washed with PBS, fixed for 30 min in 4% paraformaldehyde and permeabilized (2 min on ice) with 0.01% Triton X-100 in PBS containing 1% sodium citrate. DNA damage in the neuron-bearing coverslips was determined by TUNEL, according to the manufacturer's protocol (Roche Applied Science, Laval, PQ), with fluorescence detected using FITC-conjugated streptavidin (1:500; Invitrogen). The cell nuclei were labeled with DAPI (as above), and the coverslips were washed and mounted on glass slides with 1:1 glycerol:PBS. Cells were imaged with a 20× quartz objective on the Axioplan 2 microscope and analyzed, as above. For each cell culture (from *n *separate animals) and each experimental condition, TUNEL-positive, DAPI-labeled cells were counted in 5 microscope fields (150-200 cells/field) on 2 coverslips and averaged.

To further determine whether neurons died by apoptosis, caspase 3 activation was monitored in neuron cultures that had been exposed for 24 h to microglia, treated as above (± LPS treatment; ± an SK channel blocker). The neuron cultures in the lower Transwell™ chamber were scraped and harvested, solubilized and mixed with the fluorescent synthetic substrate, Ac-DEVD-AMC, according to the manufacturer's protocol (Calbiochem). After the supernatant was transferred to 24-well black-walled plates (PerkinElmer Life & Analytical Sciences, Woodbridge, ON), the fluorescence signal in each well was determined, and compared with the negative control (fluorogenic substrate alone). Finally, to quantify tyrosine nitration levels in the neuron-bearing coverslips, the cells were fixed (2% glutaraldehyde, 2% paraformaldehyde), transferred to 24-well black-walled plates, labeled for 18 h at 4°C with a rabbit polyclonal anti-nitrotyrosine antibody (1:200; Cell Signaling Technology, Beverley, MA), and then washed and labeled for 2 h at room temperature with a Cy3-conjugated secondary antibody (1:500; Jackson Labs).

### Standardization of plate-reader assays

A similar standardization procedure was used for TUNEL, caspase 3 activation and tyrosine nitration in neurons, and for microglial p38 MAPK activation, NFκB activation, iNOS expression and nitric oxide production. The fluorescence signal from each experimental well was measured with a plate reader (SPECTRAmax Gemini EM; Molecular Devices, Sunnyvale, CA), and then standardized as relative fluorescence units (RFU) per milligram of protein, where protein content was measured in each well with a plate reader (model EL311SX, Bio-Tek Instruments, Winooski, VT) using a colorimetric protein assay and BSA standards (Bio-Rad Laboratories, Hercules, CA). For each experiment, the negative control signal was subtracted as the average from 3 control wells lacking cells. Each experimental *(n) *value was obtained by averaging the RFU/mg protein from 3 coverslips of cells cultured from one animal; multiple *n*'s reflect cultures from different animals.

### Monitoring microglial activation

p38 MAPK activation was determined from the amount of phosphorylated p38 MAPK and NFκB activation was monitored by degradation of IκB-α using the plate reader, as before [22,42]. Microglia were seeded on coverslips in 24-well plates (200,000 cells/well) and stimulated for 30 min with 100 ng/ml LPS. The cells were washed with PBS (3×, 5 min each), fixed for 30 min in 4% paraformaldehyde, washed again (3×, 5 min each) and permeabilized for 2 min on ice with 0.01% Triton X-100. Microglia were labeled (18 h, 4°C) with a rabbit polyclonal antibody against p38 MAPK (1:750) or phospho-p38 MAPK (1:50; both from Cell Signaling) or IκB-α (1:100; Santa Cruz Biotech., Santa Cruz, CA). After further washing (PBS, 3×, 5 min each), immunoreactivity was detected using a secondary antibody (2 h, room temperature) conjugated either to Cy3 or FITC (1:500; Jackson Labs).

To monitor iNOS and nitric oxide, microglia were plated at 50,000 cells/well in a 96-well plate, cultured overnight (37°C; 5% CO_2_), and then stimulated for 24 h with LPS in the presence or absence of an SK blocker. iNOS induction was monitored with a mouse monoclonal antibody (1:200; Cell Signaling) and a Cy3-conjugated secondary antibody (1:500; Jackson Labs). The fluorescence intensity in each well was standardized to protein concentration (as above), with background subtraction using control wells lacking the primary antibody. Nitric oxide in supernatants harvested 24 h after LPS stimulation was measured using the colorimetric Griess assay (Invitrogen). Ten microliters of 0.1% N-(1-naphthyl) ethylenediamine and 10 μl of sulfanilic acid were added to 100 μl of cell supernatant from each well. The red product from the reaction with nitrite was monitored with a spectrophotometric plate reader (EL311SX, Bio-Tek Instruments, VT) as the absorbance at 570 nm and the nitric oxide concentration was calculated by interpolation on a standard curve.

For phagocytosis, we used a fluorometric assay [43] in which 100 μl of a suspension of heat-killed fluorescein-conjugated *E. coli *K-12 bacteria (Invitrogen) was added to each well of a 96-well plate containing 50,000 microglia, and incubated with or without 100 nM apamin. Following incubation (1 h, 37°C, 5% CO_2_), 50 μl of Trypan blue was added to quench the fluorescence of any *E. coli *adhering to the outside of the microglia. The fluorescence signal in each well was measured at 480 nm excitation and 520 nm emission wavelengths, and is proportional to the number of FITC-conjugated *E. coli *phagocytosed by the microglia. As a negative control, we blocked phagocytosis with 10 μM of the actin polymerization inhibitor, cytochalasin D, and compared the signal with solvent alone (0.2% DMSO).

### Statistical analyses

Where appropriate, data are presented as the mean of each treatment group ± the standard error of the mean (SEM). Statistical analyses were performed using Origin 7.0 software (Microcal, CT). Unless otherwise specified, ANOVA followed by the Bonferroni post-hoc test was used to assess the statistical significance of differences between groups.

## Results

### Expression of *KCNN*/SK channels in microglia

**Figure 1:**
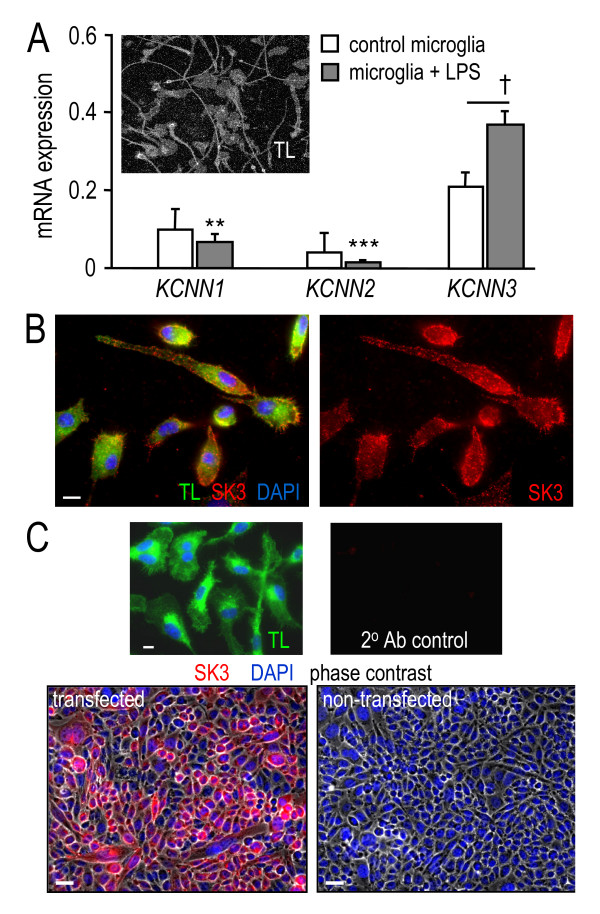
***KCNN*/SK expression in cultured microglia**. **A**. Relative mRNA expression of *KCNN1*-*KCNN3 *was determined by quantitative real-time RT-PCR (qRT-PCR), and normalized to the housekeeping gene, TATA box-binding protein (TBP). Bars represent mean ± SEM for 3 cultures from separate rat litters. After microglia were activated with lipopolysaccharide (LPS; 100 ng/ml, 24 h), *KCNN3 *expression increased (^†^*p *< 0.5) and was higher than *KCNN1 *and *KCNN2 *expression (**p < 0.01; ***p < 0.001). The inset shows a typical, essentially pure microglial culture labeled with FITC-conjugated tomato lectin. **B**. SK3 immunostaining in unstimulated microglia. Cultured microglia were labeled with anti-SK3 (rabbit polyclonal, 1:200), a Cy3-conjugated secondary (donkey anti-rabbit, 1:400; red), the microglia/macrophage stain, FITC-conjugated tomato lectin (1:500; green) and the nuclear marker, DAPI (1:3000; blue). Scale bars, 10 μm. **C**. Specificity of the SK3 staining. Upper panels: Lack of non-specific staining (no SK3 primary antibody) in microglia, which were labeled with DAPI (blue), tomato lectin (green) and the Cy3-conjugated secondary antibody (left). The color-separated image at the right shows the absence of non-specific staining in the Cy3 (red) channel. Scale bar, 10 μm. Lower panels: Phase-contrast images show SK3 staining in transfected (left), but not in non-transfected CHO cells (anti-SK3 and Cy3-conjugated secondary antibodies, as in panel B). Scale bars, 30 μm.

[For all quantitative data, statistical analyses and levels of significance are shown on the bar graphs.] Figure 1A shows transcript expression of three *KCNN *channels in cultured microglia. The cultures were ≥99% pure, as shown here by labeling with tomato lectin, and as previously shown with real-time RT-PCR and staining with OX-42 or anti-Iba1 antibodies [22,29,30]. All three genes were expressed in microglia, but after stimulation with lipopolysaccharide (LPS), *KCNN3 *expression increased ~1.7 fold and was significantly higher than *KCNN1 *and *KCNN2*. There was punctate SK3 immunoreactivity inside and at the microglial cell surface (Figure 1B). We had previously validated this antibody by Western blots on microglia and rSK3-transfected CHO cells [21]; Figure 1C shows specific immunolabeling of microglia and rSK3-transfected CHO cells, but no labeling of non-transfected cells. This verification was important for the *in vivo *analysis presented below. Microglial morphology is highly variable in culture; we could not quantify changes in SK3 labeling after LPS-induced activation because the processes retracted and cell bodies enlarged.

### Blocking microglial SK3 channels reduces their neurotoxic capacity

Effects of blocking SK channels specifically in microglia could only be studied *in vitro *because SK channels are also expressed in neurons and astrocytes (see Discussion). Roles of microglial SK channels were determined using a two-chamber Transwell™ system in which only the microglia were exposed to channel blockers. Effects of apamin on microglia are most likely due to SK3 channel block because *KCNN2 *was nearly undetectable in LPS-activated microglia, and rodent *KCNN1 *apparently does not form functional channels [44,45]. Nevertheless, we used a subtractive pharmacological approach to definitively implicate SK3. Each experiment followed the same format: if 100 nM apamin (blocks SK1, SK2, SK3) had an effect, then 5 nM tamapin (blocks SK2, SK3) was tested; if effective, then 250 pM tamapin (blocks only SK2) was tested. A specific role for SK3 was deduced if a function was inhibited by apamin and 5 nM tamapin only. Conversely, inhibition by 250 pM tamapin would implicate SK2 channels.

**Figure 2:**
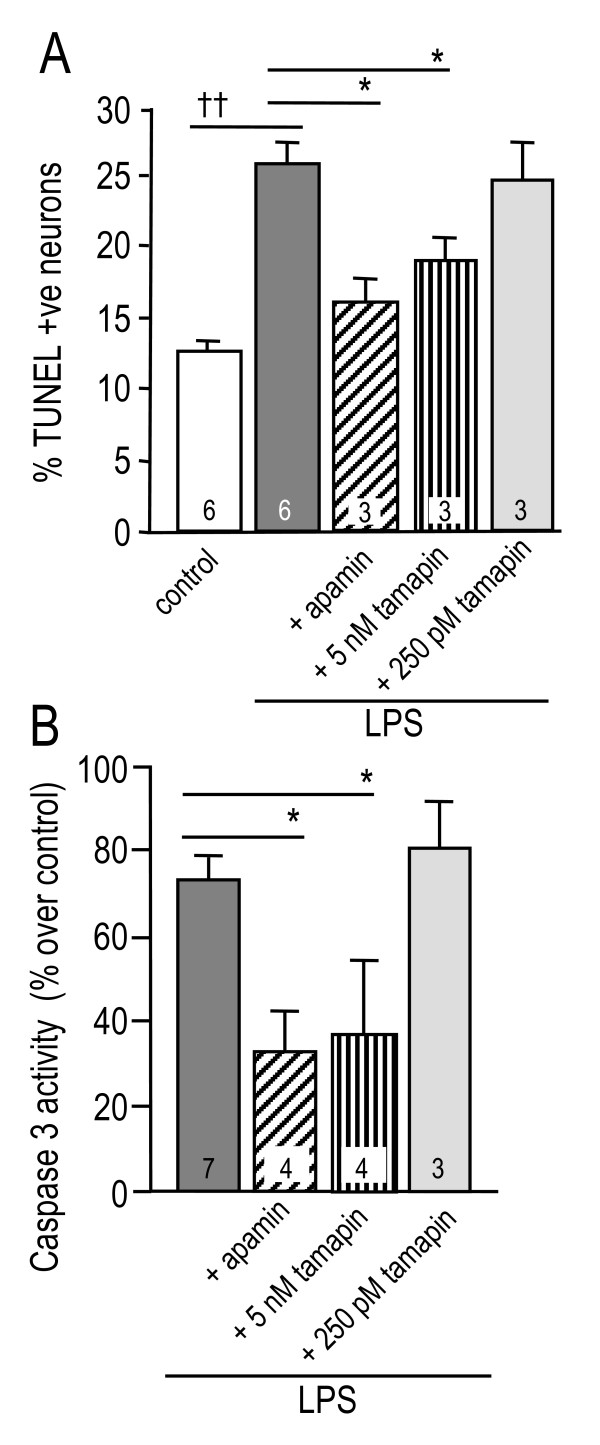
**Blocking SK3 channels in microglia reduces their neurotoxic behavior**. Microglia on Transwell™ inserts were incubated with lipopolysaccharide (LPS; 100 ng/ml, 24 h), with or without 100 nM apamin, 5 nM tamapin or 250 pM tamapin. The inserts were then washed to remove the drugs; thus, target neurons were never exposed to LPS or channel blockers. Each microglia-bearing insert was placed in a Transwell™ chamber above healthy neurons, incubated for 24 h (for caspase 3 activation) or 48 h (for TUNEL), and then the target neuron cultures were removed and assessed. Results are presented as mean±SEM for the number of separate cultures indicated on the bars. **A**. TUNEL-positive neuronal nuclei were counted and expressed as a percentage of all DAPI-stained nuclei. LPS-stimulated microglia killed more neurons than untreated microglia (^††^*p *< 0.01), and killing was significantly reduced by treating the microglia with 100 nM apamin (*p < 0.05) or 5 nM tamapin (*p < 0.05), but not with 250 pM tamapin. **B**. Average caspase 3 activity in each neuron-containing well was measured with a fluorogenic substrate (Ac-DEVD-AMC) and the fluorescence plate reader. Treating microglia with either 100 nM apamin or 5 nM tamapin reduced caspase-3 activation in the target neurons (*p < 0.05); whereas, 250 pM tamapin treatment had no effect.

The ability of microglia to kill neurons was determined using cultures that were ~70% neurons (based on MAP-2, a marker of mature neurons) and 30% astrocytes (stained with glial fibrillary acidic protein). The cultures were derived from whole rat brains, and thus were a mixed neuronal population. After exposure to microglia in the Transwell™ chambers, DNA damage in the target neuron/astrocyte cultures was quantified by counting TUNEL-positive cells as a percentage of DAPI-labeled cells in each well. The damaged cells were neurons, as they were GFAP-negative, and this is consistent with our earlier finding that LPS-activated microglia do not kill astrocytes during the 48 h treatment period [22]. After exposure to activated microglia, 26 ± 2% of the target neurons became TUNEL-positive (Figure 2A), which was significantly higher than in wells exposed to unstimulated microglia (13 ± 1%). Less than 1% of neurons died in control wells lacking microglia. Treating activated microglia with 100 nM apamin reduced the number of TUNEL-positive neurons to 16 ± 1%, while 5 nM tamapin reduced it to 19 ± 1%. There was no effect of 250 pM tamapin. Very similar results (no figure) were obtained when TUNEL fluorescence was measured with the plate reader; i.e., activated microglia nearly doubled neuron TUNEL, and this was reduced ~64% by 100 nM apamin and ~85% by 5 nM tamapin, but was unaffected by 250 pM tamapin. To further assess whether neuron death was apoptotic, caspase 3 activity was monitored in the target neuron/astrocyte cultures. Entirely consistent results were obtained (Figure 2B); activated microglia increased caspase 3 activity in neuron/astrocyte cultures by ~70%. This was reduced ~58% by 100 nM apamin and ~52% by 5 nM tamapin, but 250 pM tamapin had no effect. Together, these results show that blocking SK3 channels in activated microglia reduces their ability to kill neurons by apoptosis.

### SK3 channels contribute to a subset of microglial functions

**Figure 3:**
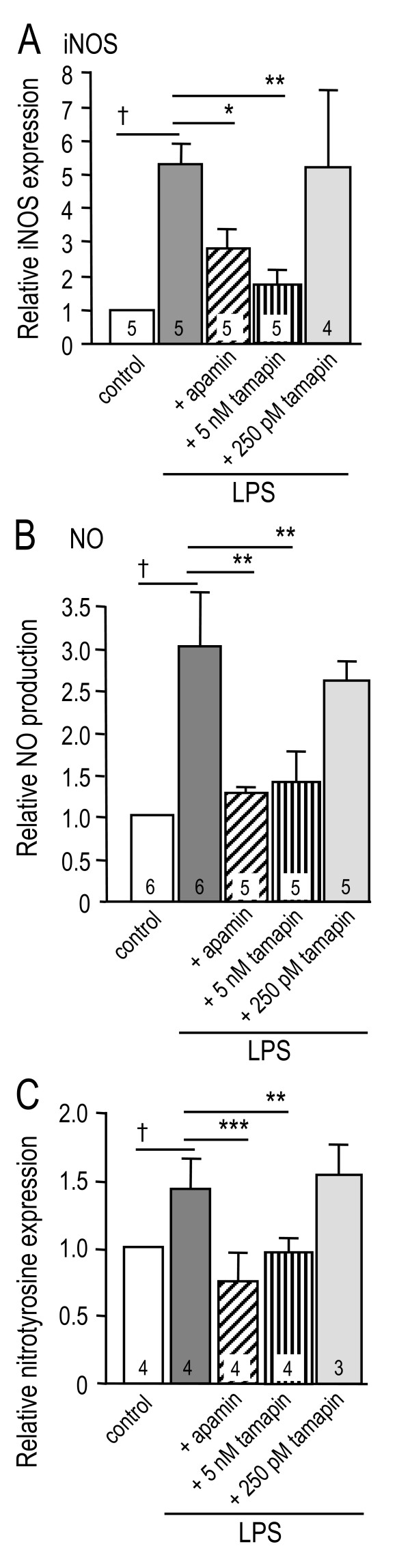
**Blocking SK3 channels reduces microglial iNOS and NO, and tyrosine nitration of target neurons**. Microglia were grown on Transwell™ inserts, and treated with LPS (100 ng/ml, 24 h), with or without 100 nM apamin, 5 nM tamapin or 250 pM tamapin. Results are expressed as RFU/mg protein in each well (mean±SEM; # of cell cultures indicated on bars), normalized to the signal from untreated microglia. **A**. iNOS protein was monitored with a mouse monoclonal anti-iNOS antibody (1:200) and a Cy3-conjugated secondary antibody (1:500). LPS stimulation increased iNOS expression (^†^*p *< 0.05), which was reduced by 100 nM apamin (*p < 0.05) or 5 nM tamapin (**p < 0.01), but not by 250 pM tamapin. **B**. Nitric oxide production was measured as nitrite accumulation, using the Griess assay. LPS increased NO production (^†^*p *< 0.05), which was abrogated by 100 nM apamin (**p < 0.01) or 5 nM tamapin (**p < 0.01), but unaffected by 250 pM tamapin. **C**. Tyrosine nitrated proteins in the target neurons were monitored with a rabbit polyclonal antibody against nitrotyrosine residues (1:200) and Cy3-conjugated secondary antibody. LPS-stimulated microglia induced tyrosine nitration in neurons (^†^*p *< 0.05), and this was abrogated by treating the microglia with 100 nM apamin (***p < 0.001) or 5 nM tamapin (**p < 0.01), but not by 250 pM tamapin.

**Figure 4:**
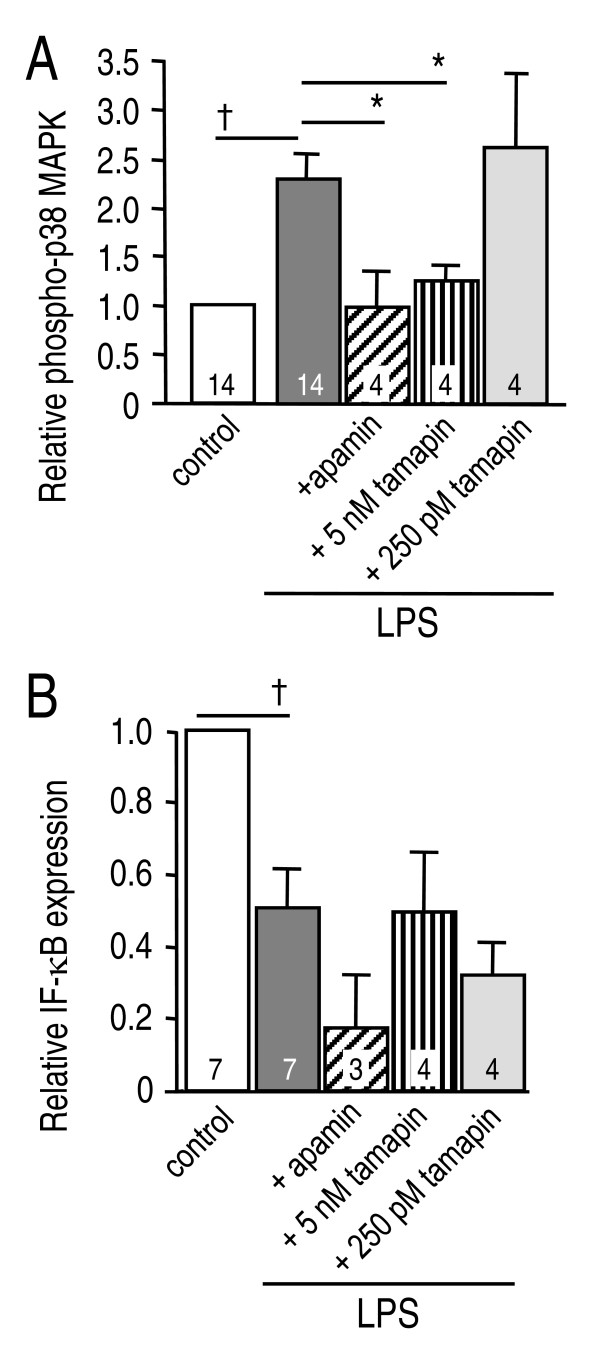
**Blocking microglial SK3 channels inhibits p38 MAPK (but not NFκB) activation**. Microglia cultures were treated with LPS (100 ng/ml), with or without 100 nM apamin, 5 nM tamapin or 250 pM tamapin. p38 MAPK activation was monitored with a rabbit polyclonal antibody against phosphorylated (active) p38 MAPK (1:50). NF-κB activation was monitored as degradation of IκB-α, using a rabbit polyclonal antibody against IκB-α (1:100). Immunoreactivity was detected using a Cy3-conjugated secondary antibody (1:500). **A**. The phospho-p38 MAPK fluorescence signal was increased in microglia after 30 min lipopolysaccharide treatment (†*p *< 0.05). This activation was prevented by 100 nM apamin (*p < 0.05) or 5 nM tamapin (*p < 0.05), but not by 250 pM tamapin. **C**. NF-κB was activated in microglia after 30 min lipopolysaccharide treatment, as judged by the decrease in IκB-α fluorescence (^†^*p *< 0.05). NF-κB activation was not affected by apamin or tamapin.

**Figure 5:**
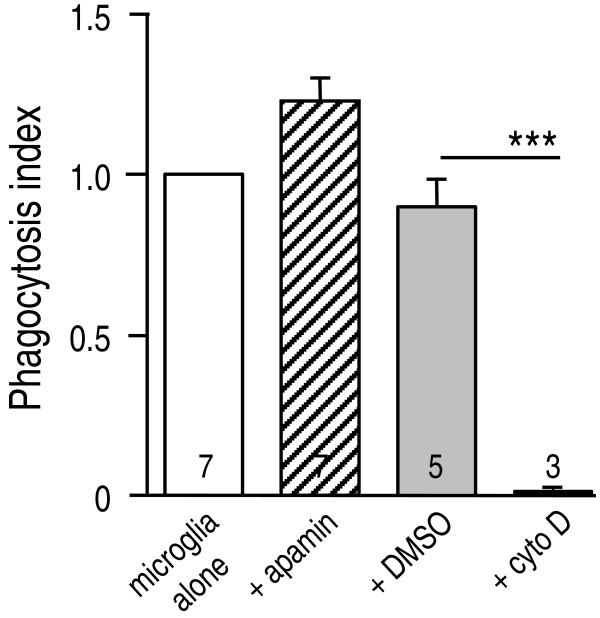
**The SK blocker, apamin, does not affect microglial phagocytosis**. Phagocytosis of fluorescein-labeled *E. coli *bacteria was quantified using a fluorescence plate reader (see Methods), expressed as mean±SEM of the number of cell cultures indicated on each bar. To generate a phagocytosis index, data were normalized to the mean fluorescence signal from untreated microglia that had phagocytosed *E. coli*. Phagocytosis was not affected by a high concentration of apamin (100 nM) that blocks cloned SK1, SK2 and SK3 channels. In control experiments, phagocytosis was prevented by the actin-polymerization inhibitor, 10 μM cytochalasin D (***p < 0.001) compared with its solvent, 0.2% DMSO. The signal from DMSO treated microglia did not differ from untreated cells (*p *= 0.50).

In Figures 3, 4 and 5, plate-reader results are expressed as relative fluorescence units per milligram of protein (i.e., RFU/mg) in each well (mean±SEM; # of microglial cultures indicated on bars). Statistical analyses were performed on the raw data but for presentation, fluorescence values were normalized to untreated controls, which were set to 1.0.

In activated microglia, iNOS increased more than 5 fold; this induction was inhibited ~58% by 100 nM apamin and ~83% by 5 nM tamapin, but was not affected by 250 pM tamapin (Figure 3A). Once induced, iNOS is enzymatically active and produces nitric oxide (NO). NO production was increased ~3 fold; i.e., from 19 ± 5 nmoles/mg protein in control cells to 59 ± 11 nmoles/mg after LPS stimulation. This increase was prevented by 100 nM apamin, reduced ~85% by 5 nM tamapin, but unaffected by 250 pM (Figure 3B). Using a similar model of LPS-activated microglia, we previously showed that either an iNOS inhibitor (S-methylisothiourea) or a peroxynitrite scavenger (FeTmPyP) greatly reduced neuron apoptosis [22,42]. Because one potential outcome of peroxynitrite formation is nitration of cell proteins, we asked whether blocking the SK3 channel in microglia reduces tyrosine nitration in the target neuron cultures. Activated microglia evoked a 160% increase in tyrosine nitration, and this was reduced 50% by 100 nM apamin, 33% by 5 nM tamapin, but was unaffected by 250 pM tamapin (Figure 3C). Taken together, our results show a role for SK3 channels in iNOS induction and NO production by microglia, and in tyrosine nitration of neuronal proteins. They do not, however, prove that peroxynitrite caused the tyrosine nitration and neuron death.

Microglia stimulation by LPS rapidly activates p38 mitogen activated protein kinase (p38 MAPK) and nuclear factor-κB (NF-κB), which we routinely detect after 30 min by Western blot and spectrofluorimetric analyses [22,42]. Microglial activation induced a ~230% increase in microglial p38 MAPK activation, detected as an increase in its phosphorylated form after 30 min (Figure 4A). p38 MAPK activation was essentially abolished by 100 nM apamin, was reduced ~80% by 5 nM tamapin, but was not affected by 250 pM tamapin. NF-κB activation was quantified as degradation of the 'inhibitory factor κB' (IκB-α), as before [22]. NF-κB was activated by LPS, as seen by the ~57% reduction in IκB-α protein after 30 min (Figure 4B). SK channels were not involved in NF-κB activation because neither apamin nor tamapin affected IκB-α degradation. These results implicate SK3 channels in a specific microglial activation pathway: p38 MAPK activation.

An important question is whether SK3 blockade impairs beneficial functions. We found that rat microglia phagocytosed fluorescent-labeled heat-killed *E. coli*, but this crucial function was not affected by 100 nM apamin (Figure 5), which demonstrates that functional SK1-SK3 channels are not necessary. In control experiments, the fluorescence signal was nearly abolished by inhibiting phagocytosis with the actin polymerization inhibitor, cytochalasin D; indicating a lack of stray fluorescence from adherent *E. coli*. Together with the lack of NFκB inhibition, this result also demonstrates that apamin was neither toxic nor a generalized microglial suppressant.

### SK3 immunoreactivity *in vivo *before and after a hemorrhagic or ischemic stroke

**Figure 6:**
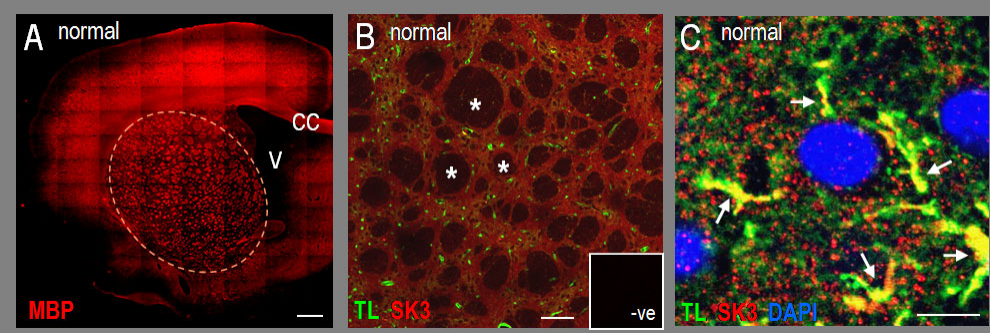
**SK3 immunoreactivity in the healthy adult rat striatum**. Confocal micrographs of coronal sections show salient features of the adult rat striatum, microglia/macrophages, and SK3 staining. **A**. A low-magnification image of the entire hemisphere (scale bar, 1 mm); the striatum is indicated by a dashed curve; V, ventricle; CC, corpus callosum. White matter is labeled with an antibody against myelin basic protein (MBP) (mouse monoclonal, 1:100) and a Cy3-conjugated secondary antibody (donkey anti-mouse, 1:400); note the dark 'holes' between the brightly stained axon bundles seen in cross-section. **B**. Diffuse SK3 staining is seen between the unstained white matter tracts (asterisks) in the normal striatum: co-labeled with FITC-conjugated tomato lectin (TL, 1:400), and anti-SK3 (rabbit polyclonal, 1:500) with a Cy3-conjugated secondary antibody (donkey anti-rabbit, 1:400); scale bar, 100 μm. Lack of non-specific staining is shown by the negative control (inset) with Cy3-conjugated 2° antibody alone. **C**. A high-magnification image (scale bar, 5 μm) shows intense SK3 staining in microglial processes (marked with arrows) and punctate staining elsewhere (labeled as above; and with the nuclear stain, DAPI).

Having found that SK3 channels contribute to microglial activation *in vitro*, it was important to demonstrate SK3 expression in microglia *in vivo*, particularly in the damaged CNS. We examined SK3 immunoreactivity both in the healthy adult rat striatum and during the pronounced inflammatory phase after either an intracerebral hemorrhage (ICH) or ischemic stroke. For orientation, Figure 6A shows the structure of the striatum, with myelin basic protein-labeled axon bundles seen in cross section. SK3 immunoreactivity (Figure 6B) was diffuse and widespread between the unstained white matter tracts. When examining SK3 immunoreactivity, microglia were labeled with tomato lectin, which produced the clearest cell staining when combined with the polyclonal SK3 antibody. This was especially useful at higher magnifications, where cell size and morphology allows clear discrimination between these immune cells and microvessels (brain endothelial cells also stain with tomato lectin). A high-magnification image from the healthy striatum (Figure 6C) shows pronounced SK3 labeling in the processes of a resting microglial cell, and punctate staining elsewhere, which is consistent with the diffuse SK3 staining seen at low magnification in panel B.

**Figure 7:**
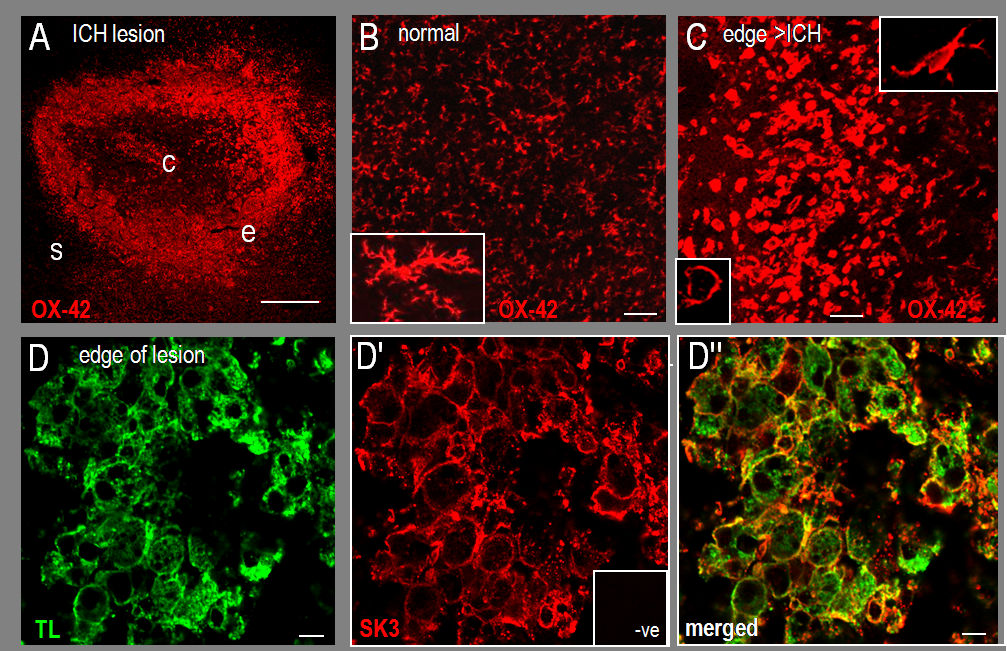
**Changes in SK3 immunoreactivity and microglia/macrophages after an intracerebral hemorrhage (ICH)**. Confocal images in A-C were labeled with OX-42 antibody (mouse monoclonal; 1:200) and a Cy3-conjugated secondary (donkey anti-mouse, 1:400). **A**. A ring of activated microglia/macrophages surrounds the hematoma at 7 days after an ICH: c, centre; e, edge; s, surrounding striatum (scale bar, 500 μm). **B**. In the normal striatum, there is widespread OX-42 labeling of 'resting' microglia (scale bar, 100 μm). The inset shows a typical ramified microglial cell with long processes and a small cell body (scale bar, 20 μm). **C**. At the edge of the hematoma 7 days after an ICH, microglia/macrophages transition to amoeboid cells with partially retracted processes further from the lesion (and inset at upper right) to round cells near the hematoma (and left inset). Scale bars: 100 μm for the main image and 20 μm for the insets. **D**. At the edge of the hematoma, SK3 labeling in microglia/macrophages (as in Figure 6) is shown in high-magnification color-separated images (scale bars, 10 μm). The negative control (inset in *D'*) shows lack of staining with Cy3-conjugated 2° antibody alone. The merged image *(D'') *shows extensive surface SK3 staining in activated microglia/macrophages (examples marked with arrows).

Figure 7 illustrates the changes in microglia morphology and SK3 staining after an intracerebral hemorrhage (ICH) was induced by injecting collagenase into the striatum. OX-42 antibody clearly labels resting and activated microglia, as well as macrophages that have infiltrated from the blood. When microglia become highly activated *in vivo*, they round up and cannot be distinguished from macrophages; thus, we use the term 'microglia/macrophages'. At 7 days after the ICH, the lesion is surrounded by a ring of activated microglia/macrophages (Figure 7A), as we previously showed with several labels (OX-42, Iba-1, tomato lectin, ED-1) [31-35]. For comparison, microglia in the undamaged contralateral striatum have the typical highly ramified 'resting' morphology (Figure 7B). The edge of the hemorrhagic lesion (Figure 7C) shows the transition from 'ameboid' microglia with short protrusions (high-magnification inset at right) to large round cells (≥20 μm in diameter; left inset). Intense SK3 immunoreactivity and considerable surface labeling was seen in activated microglia/macrophages, as shown in a high-magnification color-separated image (Figure 7D) from the dense cellular band at the edge of the lesion.

**Figure 8:**
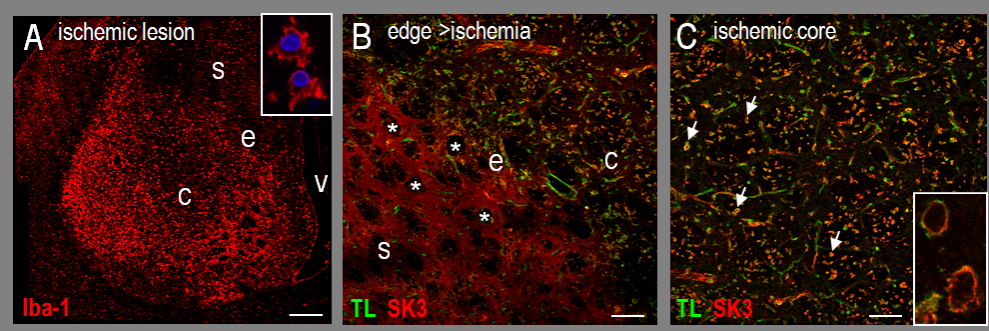
**SK3 immunoreactivity in the striatum 3 days after an ischemic stroke. A**. The ischemic lesion (scale bar, 500 μm) shows increased staining with the microglia/macrophage marker, 'ionized calcium-binding adapter-1' (Iba-1; rabbit polyclonal, 1:1000; Cy3-conjugated donkey anti-rabbit secondary, 1:400). The high-magnification image from the core (inset; scale bar, 20 μm) shows examples of activated microglia/macrophages, co-labeled with the nuclear stain, DAPI. **B, C**. Sections from the edge and centre of the lesion were co-labeled with tomato lectin and anti-SK3 (as in Figure 6). Scale bars: 100 μm for the main images; 20 μm for the inset. In panel B, note the unlabeled white matter tracts at the edge of the lesion (asterisks) surrounded by areas of diffuse SK3 staining. In panel C, almost every activated microglia/macrophage (examples marked with arrows) is co-labeled with SK3 and tomato lectin. Note the intense surface SK3 labeling of activated microglia/macrophages (inset).

Similar changes in microglia/macrophages and SK3 immunoreactivity were seen after an ischemic stroke was induced by injecting endothelin-1 into the striatum. A low-magnification image (Figure 8A) illustrates the ischemic lesion using an antibody against 'ionized calcium-binding adapter-1' protein (Iba-1). Increased Iba-1 labeling of immunoreactivity microglia and macrophages is a hallmark of stroke-induced inflammation, while labeling is less intense in the surrounding striatum [37]. The high magnification inset shows images of activated microglia/macrophages from within the lesion. An image from the edge of the infarct (Figure 8B) illustrates the pronounced changes in SK3 immunoreactivity. In the relatively undamaged surrounding striatum at the lower left, the distribution of SK3 is the same as in the undamaged striatum (compare with Figure 6B), including diffuse SK3 staining between white matter tracts. In contrast, within the lesion, discrete cellular SK3 staining is seen in activated microglia/macrophages. At the center of the infarct (Figure 8C), SK3 staining is largely restricted to activated microglia/macrophages, which is especially clear in the high magnification inset. Note the loss of diffuse punctate staining outside these cells after both forms of stroke.

## Discussion

Previous studies of SK channels in the CNS have emphasized their expression and roles in neurons. Transcripts for *KCNN1*, *KCNN2 *and *KCNN3 *have been detected in the CNS by Northern analysis, *in situ *hybridization and RT-PCR, and immunoreactivity has been shown in some neurons [24,26,46]. Because the *KCNN3*/SK3 channel is being considered as a therapeutic target, it is crucial to understand its expression and roles in both neuronal and non-neuronal CNS cells. SK3 is preferentially expressed in phylogenetically older brain regions (thalamus, basal ganglia, cerebellum, brainstem) [47-50]; for instance, *KCNN3 *mRNA [47] is relatively high in the caudate putamen and in dopaminergic neurons of the substantia nigra, and SK3 immunoreactivity [49] is present in the caudate putamen. Reports of SK3 expression in non-neuronal cells are very limited; it is expressed in astrocytes in the supraoptic nucleus and in olfactory ensheathing glial cells [51,52], and it contributes to vasodilation in response to endothelium-derived hyperpolarizing factor (EDHF) [53]. Here, we observed widely distributed and punctate SK3 immunostaining in the healthy adult rat striatum (except in white matter tracts), and for the first time, have shown SK3 immunoreactivity in microglia *in vivo *and *in vitro*. The isolated microglia were from neonatal rats and the *in vivo *studies were from adults; thus, SK3 is apparently expressed in microglia throughout post-natal development. The punctate nature of SK3 staining in the brain has not been addressed previously. It can be partly accounted for by the staining pattern in microglia, which was punctate both *in vivo *and *in vitro*. SK3 was co-localized with markers of presynaptic terminals in an *in vitro *analysis of cultured hippocampal neurons [54], and if this occurs *in vivo *and in striatal neurons, it would also appear punctate.

Changes in SK3 expression have not been examined in the damaged CNS. We observed pronounced changes in staining patterns of activated microglia/macrophages after either a hemorrhagic or ischemic stroke. Microglia undergo complex 'activation' processes in response to CNS damage or disease, which correspond with pronounced changes in morphology (illustrated in Figure 7). Activated microglia/macrophages formed a dense band of cells at the edge of the hemorrhagic lesion (other cells die inside the hematoma) [31-35], creating a zone where cellular SK3 labeling was easily distinguished. When viewed at high magnification, essentially every microglia/macrophage in this zone showed strong SK3 immunoreactivity, including substantial staining at the cell surface. After an ischemic stroke, similar ring-like SK3 staining was seen in activated microglia/macrophages within the infarct (see Figure 8). Another striking change was the loss of neuronal SK3 staining in the ischemic infarct. Neuron cell bodies and axons are still present at this time after the stroke [37]; hence, this provides novel evidence that neuronal SK3 expression changes after acute CNS damage.

For microglia, which are considered non-excitable cells (but see [55]), SK channels are well designed to contribute to cellular functions because channel gating is voltage independent and only a small elevation in free Ca^2+ ^(K_d _~300 nM) is required to activate them [18,26]. Such Ca^2+ ^increases in microglia are readily achievable through any of several receptor-ligand interactions, ion channels and transporters, including purinergic receptors, store-operated Ca^2+ ^entry [17,27], CRAC channels [30] and reversed Na^+^/Ca^2+ ^exchange [56]. Importantly for this study, lipopolysaccharide (LPS) increases Ca^2+ ^in microglia and activates p38 MAPK and NF-κB, up-regulates pro-inflammatory molecules [17,27,28,57] and increases their capacity to kill neurons through a peroxynitrite-mediated mechanism [22,42,58]. By using potent peptidyl blockers of SK channels we demonstrated important new links between SK3 channels (but not SK1 or SK2) and microglial activation through p38 MAPK, without a role in NF-κB activation. Of note, p38 MAPK contributes to iNOS and NO production [59-63], and p38 MAPK inhibitors are being tested for stroke [61,63]. Very little is known about the involvement of ion channels in microglial phagocytosis, except for our recent report that swelling-sensitive Cl^- ^channels play a role [43]. Here, our observation that apamin-sensitive SK channels are not required for bacterial phagocytosis might reflect a lack of channel activation because phagocytosis can be Ca^2+ ^independent [64]. This insensitivity to SK channel block is important because phagocytosis is an essential microglial function that clears necrotic and apoptotic cells, which otherwise could increase inflammation, provide a source of antigens and evoke autoimmune responses [10,11].

Intriguingly, simultaneous involvement of apamin-sensitive SK channels and *KCNN4*/KCa3.1 has been seen in some cellular functions; e.g., in blood vessels [65,66], and in microglia, where we also find that the TRAM-34-sensitive KCa3.1 channel [22] contributes to microglial activation and neurotoxicity. This convergence is not a failure of blocker specificity, as SK1-SK3 channels are the only known targets for apamin, and TRAM-34 is specific for *KCNN4*/KCa3.1 [26,67]. No explanations have been offered in the literature for the need for two types of Ca^2+^-activated K^+ ^channels in any cell in which they co-exist. While recognizing that these ideas are speculative, we present some possibilities for future investigation. *(i) *There is one report that SK3 and KCa3.1 form homotetramers in rat brain [49], but because they can interact in expression systems [68], their native tetrameric composition should be further examined. *(ii) *In neurons, different KCa channels can couple to different sources of intracellular Ca^2+ ^[69], possibly in microdomains comprised of different macromolecular signaling complexes [25,70]. Microglia also have multiple Ca^2+ ^stores and entry pathways [17], and because nothing is known about their coupling to SK3 and KCa3.1 channels, this should be examined. *(iii) *Our finding that both SK3 and KCa3.1 regulate p38 MAPK activation and nitric oxide production might reflect a convergence of signaling. Tyrosine phosphorylation regulates p38 MAPK activation [71] and iNOS induction [72], and LPS activates the p38α isoform of MAPK [71,73], which interacts with 'nuclear activator protein-1' to induce iNOS transcription [74]. *(iv) *Both SK3 and KCa3.1 channels respond to similar rises in Ca^2+^, but they could become activated at different times if their subcellular locations differ or they are modulated by different second messengers. Trafficking of both channels is similarly regulated by the calmodulin binding domain [75,76] and other post-translational regulators of trafficking are now being addressed [77]. Nothing is known about the relative numbers of active channels in cells in which both channels are expressed.

### Broader implications

Previously, interest in SK channels in the CNS was centered on their roles in neuron excitability and their potential as therapeutic targets. High SK3 levels in dopaminergic midbrain neurons [reviewed in [26,47,78]], and its role in producing a medium-duration after-hyperpolarization (mAHP) that regulates rhythmic firing in the normal caudate putamen/striatum [79] have made it an attractive target for Parkinson's and schizophrenia. Although controversial, several studies have correlated trinucleotide polymorphisms in SK3 with schizophrenia and migraine [80,81], and changes in SK3 expression with sleep apnoea, sudden infant death syndrome, mood disorders, epilepsy and schizophrenia [24,26,46,82]. SK3 channels also regulate neuron firing in the dorsal motor nucleus and superior cervical ganglion neurons [26] and, in the hippocampus, the SK3 current increases with age and correlates with age-dependent deficits in synaptic plasticity and learning [24]. Clearly, the presence and roles of SK3 in normal microglia, as well as changes in other CNS cell types after damage must be considered. Although this study examined SK3 in the striatum with or without ischemic or hemorrhagic strokes, we have also observed strong SK3 immunoreactivity in activated microglia/macrophages in the injured cortex, retina and spinal cord (unpublished results). Thus, contributions of SK3 to microglia functions are likely to extend to other CNS areas, including regions where SK3 is lacking in neurons. The observation that SK3 contributes to p38 MAPK activation in microglia might extend to other cell types in the CNS and elsewhere. To progress further with *in vivo *functional studies, blockers and modulators must be developed that can enter the CNS and discriminate between SK2 and SK3 channels. With increased understanding of the expression and roles of SK3 in non-neuronal CNS cells, the possibility of non-neuronal effects must be thoroughly evaluated.

## Conclusions

There are several salient findings in this study. *(i) *Transcripts for all three cloned SK channels (*KCNN1*, *KCNN2*, *KCNN3*) are expressed in microglia, but *KCNN3 *predominates and is the only channel up-regulated in LPS-activated microglia. *KCNN2 *expression in activated microglia was especially low; near the limits of detection. *(ii) *There was considerable SK3 immunoreactivity throughout the healthy adult striatum *in vivo *(except in the white matter tracts), and in microglia *in vitro*. *(iii) *During the inflammatory phase after either an ischemic or hemorrhagic stroke, SK3 was especially prevalent in activated microglia/macrophages. Neuronal staining was markedly decreased in the ischemic infarct. (Neuronal changes after hemorrhage were not assessed.) *(iv) *SK3 channels (but not SK1 or SK2) contributed to microglial activation, which up-regulated iNOS and NO production, and involved a functional link to signaling through p38 MAP kinase. *(v) *Blocking SK3 channels in microglia reduced their ability to induce apoptosis, caspase 3 activation and tyrosine nitration in neurons. *(vi) *Blocking microglial SK channels with a high concentration of apamin did not globally inhibit the cells; NFκB activation and phagocytosis were not inhibited. By providing evidence that targeting SK3 channels can reduce microglia-mediated neurotoxicity, this study identifies a new potential molecular target for reducing inflammation-mediated damage in acute and chronic CNS disorders.

## Abbreviations

DAPI: 4'-6-diamidino-2-phenylindole; iNOS/NOS2: inducible nitric oxide synthase; Iba-1: ionized calcium-binding adapter-1 protein; ICH: intracerebral hemorrhage; LPS: lipopolysaccharide; mAHP: medium-duration after-hyperpolarization; MEM: minimal essential medium; MBP: myelin basic protein; PBS: phosphate buffered saline; qRT-PCR: quantitative real-time RT-PCR; SK channel: small-conductance Ca^2+ ^activated K^+ ^channel; TLR4: Toll-like receptor 4

## Competing interests

The authors declare that they have no competing interests.

## Authors' contributions

LCS conceived of and supervised all aspects of the study and wrote the manuscript. VK carried out the microglial activation and neuron killing studies and accompanying statistical analysis (Figures 2, 3 and 4) and helped draft the paper. IM-E carried out the *in vivo *studies (Figures 6, 7, 8). VS carried out the phagocytosis studies (Figure 5). CV carried out the *in vitro *immunohistochemistry (Figure 1B &1C). All authors read and approved the final manuscript.
